# Alginate–Moroccan Clay, New Bio-Nanocomposite for Removal of H_2_PO_4_^−^, HPO_4_^2−^, and NO_3_^−^ Ions from Aqueous Solutions

**DOI:** 10.3390/polym15244666

**Published:** 2023-12-11

**Authors:** Rachid Aziam, Daniela Simina Stefan, Abdelali Aboussabek, Mohamed Chiban, Alexa-Maria Croitoru

**Affiliations:** 1 Laboratory of Applied Chemistry and Environment, Department of Chemistry, Faculty of Science, Ibnou Zohr University, Agadir BP 8106, Morocco; rachid.aziam@edu.uiz.ac.ma (R.A.); a.aboussabek@uiz.ac.ma (A.A.); m.chiban@uiz.ac.ma (M.C.); 2 Department of Analytical Chemistry and Environmental Engineering, Faculty of Chemical Engineering and Biotechnologies, National University of Science and Technology Politehnica of Bucharest, 1-7 Polizu Street, 011061 Bucharest, Romania; 3 Department of Science and Engineering of Oxide Materials and Nanomaterials, Faculty of Chemical Engineering and Biotechnologies, National University of Science and Technology Politehnica of Bucharest, 1-7 Polizu Street, 011061 Bucharest, Romania; alexa_maria.croitoru@upb.ro; 4 National Centre for Micro- and Nanomaterials, National University of Science and Technology Politehnica of Bucharest, 313, Spl. Independentei Steet, 060042 Bucharest, Romania

**Keywords:** bio-nanocomposite, alginate, orthophosphate ions, nitrate ions, Moroccan natural clay

## Abstract

The aim of this work is to synthesize and characterize alginate–Moroccan clay bio-composite in order to improve our understanding of the adsorption of inorganic pollutants found in textile effluents. Characterization of the bio-composite used was carried out using a variety of techniques (IR-TF, SEM, DRX, and pH_ZPC_). The influence of the medium’s physico-chemical parameters (temperature, pH, initial concentration, etc.) on the retention of inorganic pollutants was also studied. Studies of adsorption and inorganic pollutants such as orthophosphate (H_2_PO_4_^−^ and HPO_4_^2−^) and nitrate (NO_3_^−^) ions were carried out, using simple solutions from the laboratory, in a batch system. This study explored the impact of adsorbent dose, contact time, solution pH, and temperature on the adsorption process. Various kinetic models, including pseudo-first-order, pseudo-second-order, intra-particle diffusion, and Elovich models, were tested and evaluated, to illustrate the adsorption kinetics. This study’s findings demonstrated that the adsorption process follows second-order kinetics, with associated rate constants successfully determined. The correlation coefficient for the pseudo-second-order kinetic model is nearly equal to 1 (>0.98), and the value of theoretical adsorption capacity (*q_e_*_,the_) is comparable to the experimental one (*q_e_*_,the_ = 58.14 mg/g for H_2_PO_4_^−^, *q_e_*_,the_ = 54.64 mg/g for HPO_4_^2−^, and *q_e_*_,the_ = 52.63 mg/g for NO_3_^−^). Additionally, the adsorption equilibrium was investigated through the application of various mathematical models, including the Langmuir, Freundlich, Temkin, and Dubinin–Radushkevich isotherm models, to assess the mechanistic parameters associated with the adsorption process. Among these models, the Langmuir isotherm emerged as the most suitable one for characterizing the adsorption of H_2_PO_4_^−^, HPO_4_^2−^, and NO_3_^−^ ions using bio-nanocomposite beads. The maximum adsorbed amounts of metal ions by the bio-nanocomposite used were 625 mg/g for H_2_PO_4_^−^, 909.09 mg/g for HPO_4_^2−^, and 588.23 mg/g for NO_3_^−^ from the batch system. The endothermic and physical nature of the adsorption is suggested by the positive values of ΔH°, which is consistent with experimental findings. The adsorption process is spontaneous, as evidenced by the negative ΔG° values. Positive ΔS° values indicate increased randomness at the solid/liquid interface during adsorption of ion-organic ions onto the engineered bio-nanocomposite. The obtained results demonstrated that, from a scientific perspective, alginate–Moroccan clay bio-nanocomposites exhibit a highly significant adsorption capability for the removal of oxyanions in aqueous environments.

## 1. Introduction

Inorganic pollutants, particularly orthophosphate and nitrate ions, are one of the main causes of pollution. When present in high concentrations in the natural environment, they generate significant pollution, with considerable repercussions on the ecosystem and human health. 

The increasing concentration of phosphates in the environment due to human activities has been considered a matter of concern for more than four decades, but the main question is to know is the amount of the additional phosphorus that has contributed to the eutrophication of lakes, ponds, and other water bodies [1,2]. Once released into the environment, phosphate ions disperse into water bodies, mainly as a result of processes such as soil erosion, rock decomposition, and animal and wildlife droppings. 

Phosphates are one of the anions assimilated by the human body. Their presence in water is due to the release of industrial waste (food processing, laundry, and surface treatment), agricultural waste (fertilizers and pesticides), domestic waste (detergents, washing powder, and shampoos), or their use to remove corrosion and scale (polyphosphate). Phosphates are the mineral forms of phosphorus, formed from PO_4_^3−^ ions (they are generally not very soluble in water) [3,4,5,6,7]. Hydrogenated forms (H_2_PO_4_^−^ and HPO_4_^2−^) are much more soluble. 

The European Community has set a maximum contaminant level of 50 mg/L and recommended levels of 25 mg/L for industrial wastewaters [8]. In Morocco, the maximum admissible concentration values for nitrate and phosphorus in potable waters are, respectively, 50 mg/L and 0.05 mg/L, equivalent to 0.15 mg/L of hydrogen phosphate ions [8].

For agriculture and irrigation, the accepted orthophosphate standard is 0.2 mg/L [9]. An elevated concentration of nitrate in drinking water can lead to a condition in infants known as methemoglobinemia, colloquially referred to as “blue disease”, as well as other associated health issues [10]. The most important environmental problems caused by nitrogen and phosphorus compounds are eutrophication of water resources and infectious diseases [11]. The presence of orthophosphate and nitrate anions in liquid effluents poses a threat for both human health and the wider ecosystem. According to the recommendations of the World Health Organization (WHO), the permissible levels for nitrates and phosphates in water sources are established at 50 mg/L and 5 mg/L, respectively [12]. Conventional techniques for eliminating nitrate and phosphate ions from wastewater encompass methods like reduction through diverse approaches, including coagulation, flocculation [13,14], biological treatment [15], membrane filtration [3], ion exchange [16], chemical precipitation [17], and adsorption methods [13,18,19]. Due to the significant drawbacks of the existing methods for removing inorganic pollutants, adsorption is a suitable alternative for removing phosphates and nitrates from liquid effluents. Adsorption technology is unique in that it offers multiple advantages, such as low cost, high selectivity, effective ability to remove orthophosphate (H_2_PO_4_^−^, HPO_4_^2−^) and nitrate (NO_3_^−^) ions, even at low concentrations, ease of use, simple design, high capacity, and the prospect of producing minimal quantities of by-products [20,21].

Natural clays are more efficient due to their excellent swelling properties [22], low cost [23], high specific surface area [24], and other structural features [25].

Furthermore, alginate is a polysaccharide that is extracted from brown algae. It is composed of repeating units of the sugars mannuronic acid and guluronic acid, which give alginate its characteristic properties. Additionally, as a biopolymeric support for natural clay, alginate is a natural, non-toxic, low-cost, and environment friendly polymer polysaccharide with high biodegradability. Its advantageous properties, such as thickening, gelling, and stabilizing, make alginate an appealing option for researchers seeking to create composite bead adsorbents by combining it with clay [26,27]. 

Zhao et al. (2023) reported that coordination polymers, thanks to their advantageous properties such as simplicity of production, rapid response, and high sensitivity, are well suited to the detection of trace environmental toxins [28].

Elaborate bio-composites are also considered among the materials with excellent adsorption properties, in particular hybrid bio-composites based on alginate and gelatin, which are generally considered to be universal adsorbents. The formulation of bio-composite beads will pave the way for their eventual use on an industrial scale as adsorbents in water treatment, due to their ease of physical separation and reusability. Encapsulation in bio-polymer beads overcomes this problem [29]. This study focuses on the investigation of major pollutants, such as orthophosphate (H_2_PO_4_^−^ and HPO_4_^2−^) and nitrate (NO_3_^−^) ions, which are widely prevalent in this context. This work is part of a sustainable development approach using innovative, low-cost materials, with a view to implement them in aqueous effluent treatment techniques. The aim of the project is to prepare and synthesize alginate-based bio-composites, and to study the adsorption capacity of the biomaterials developed for the removal of some nitrate and phosphate ions in aqueous solutions. This study explored the impact of adsorbent dose, contact time, solution pH, and temperature on the adsorption process. Various kinetic models, including pseudo-first-order, pseudo-second-order, intra-particle diffusion, and Elovich models, were tested and evaluated to illustrate the adsorption kinetics. This study’s findings demonstrated that the adsorption process follows second-order kinetics, with associated rate constants successfully determined. The Langmuir isotherm emerged as the most suitable one for characterizing the adsorption of orthophosphate and nitrate ions using bio-nanocomposite beads. The obtained results demonstrated that, from a scientific perspective, alginate-based bio-nanocomposites exhibit a highly significant adsorption capability for the removal of inorganic pollutants in aqueous solutions.

## 2. Materials and Methods

### 2.1. Adsorbent

**Figure 1:**
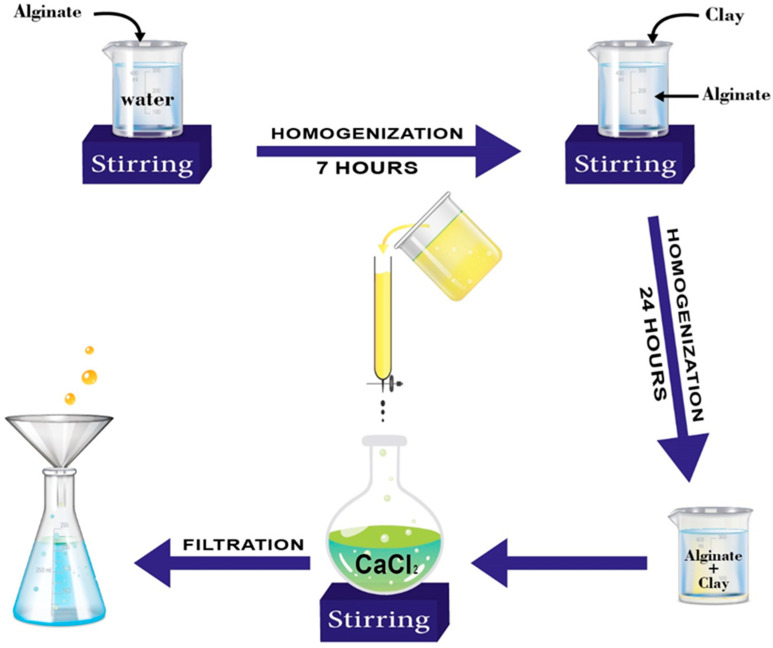
Formation of alginate–clay beads.

A bio-nanocomposite based on alginate-encapsulated Moroccan clay was developed using the extrusion synthesis method, as shown in the diagram below (Figure 1).

A mass of 1 g of alginate was continuously stirred with bidistilled water in a 100 mL Erlenmeyer flask for 7 h, at a temperature of 40 °C. The solutions were agitated at 500 rpm to completely disperse the alginate. After that, the alginate suspension received 2 g of Moroccan natural clay, while being gently magnetically stirred at room temperature. The alginate–clay solution is injected into a syringe for bead production. The syringe is held vertically above a 0.1 M calcium chloride (CaCl_2_) gelling solution. The treatment is drip-fed into the gel bath gradually. The saline solution instantly gels, and alginate beads are created by an accumulation of chains around Ca^2+^ cations. The mixture (alginate/clay beads and calcium chloride solution) is allowed to rest for a maturation time, which is sufficiently long for complete gelation. The maturation time varies depending on the cation concentration, ionic strength, and pH. After maturation, the beads are filtered and washed multiple times with distilled water. After washing, the beads are used immediately as in the case of “hydrogel” beads [29].

### 2.2. Adsorption Experiments

The adsorption experiments were conducted in batches at room temperature, except for those investigating the temperature’s impact. The batch mode was chosen for its simplicity and effectiveness. A known quantity of bio-nanocomposite beads was added to stoppered Erlenmeyer glass flasks with a 100 mL capacity, along with 50 mL of orthophosphate and nitrate ions with a known concentration and solution pH. To ensure uniform mixing, the stirring speed was maintained constant throughout each cycle. After varying the contact time (*t*), the resulting solutions were centrifuged at 5000 rpm for 10 min. The supernatant was then subjected to 0.45 μm membrane filtration, and the filtrate was analyzed. The concentration of the remaining ion solution was determined using a UV–visible spectrophotometer at its respective λ_max_ value, which is 700 nm for orthophosphate (H_2_PO_4_**^−^** and HPO_4_^2−^) ions and 415 nm for nitrates (NO_3_**^−^**). The removed orthophosphate and nitrate ion concentration (*Cr*) from the aqueous solution was calculated as the difference between the initial concentration (*C*_0_) and the concentration at different contact times. The initial pH is equal to 7.73 for the HPO_4_^2−^ solution, 5.22 for the H_2_PO_4_^−^ solution, and 6.52 for the NO_3_^−^.

The amount removed per unit mass of adsorbent (*q_t_*, mg/g) at time “*t*” was calculated as follows:

(1) $$q_{t}=\left(C_{0}-C_{t}\right)\times \frac{V}{m}$$

The removal percentage of H_2_PO_4_^−^, HPO_4_^2−^, and NO_3_^−^ ions was calculated by:

(2) $$\%adsorption=\frac{100(C_{0}-C_{t})}{C_{0}}$$

where *q_t_* (mg/g) indicates the amount of H_2_PO_4_**^−^**, HPO_4_^2−^, and NO_3_**^−^** ions removed per unit mass of the adsorbent at a given time (*t*). *C*_0_ (mg/L) represents the initial concentration of H_2_PO_4_**^−^**, HPO_4_^2−^, and NO_3_^−^ ions in the aqueous solution. *C_t_* (mg/L) is the concentration of H_2_PO_4_^−^, HPO_4_^2−^, and NO_3_^−^ ions at time *(t*). *V* (L) denotes the volume of the working solution.

## 3. Results and Discussion

### 3.1. Characterization of Bio-Nanocomposite Beads

#### 3.1.1. XRD Analysis

**Figure 2:**
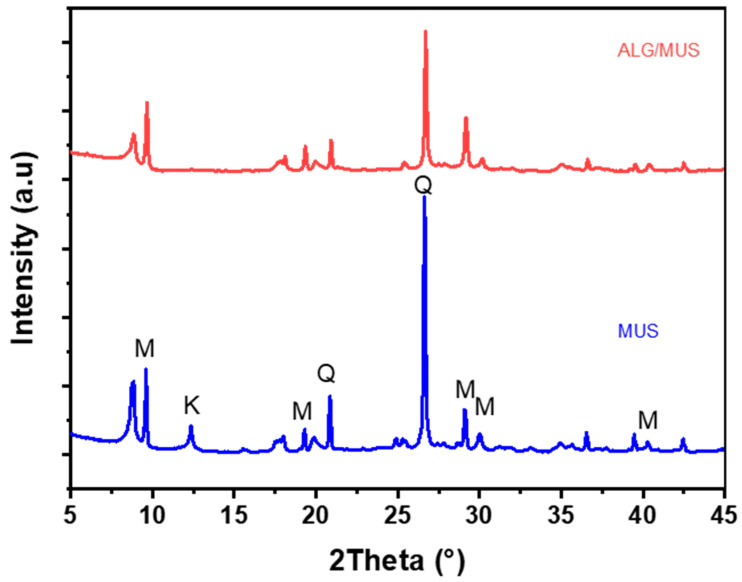
X-ray diffraction of the natural clay (MUS) and alginate–clay bio-nanocomposite (ALG/MUS).

The alginate–clay bio-nanocomposite elaborated was prepared and characterized by several analyses. The XRD technique was evaluated within the scanning range of 5° ≤ 2θ ≤ 80° to confirm the crystalline structure of alginate–clay bio-nanocomposites. The X–ray diffraction spectra of the alginate–clay bio-nanocomposite elaborated is displayed in Figure 2. The X–ray diffraction analysis was examined using a Bruker CCD–Appex (Faculty of Science, Ibnou Zohr University, Agadir, Morocco) apparatus equipped with an X-ray generator (Ni filtered Cu-K*α* radiation), operated at 40 kV and 40 mA. Samples in powder form were scanned from 5° to 80° (2θ), at a step of 2° min^−1^. The diffraction signals at 2θ values of 9.61°, 18.05°,19.85°, 29.13°, 35.03°, and 42.45° correspond to the lattice planes of clay (Muscovite) mineral. Additionally, there is a diffraction peak at 2θ = 20.95°, 26.68°, and 42.60°, indicating the presence of quartz [30,31]. The diffractograms of alginate–clay bio-nanocomposites demonstrate the effective dispersion of clay layers within the amorphous alginate matrix (ALG). This dispersion is evident from the observed shifts and reduction in the intensity of the peaks, which are typically associated with the interbasal distances between the clay layers [29,32].

#### 3.1.2. pH of Zero point charge for Bio-Nanocomposite Adsorbent

**Figure 3:**
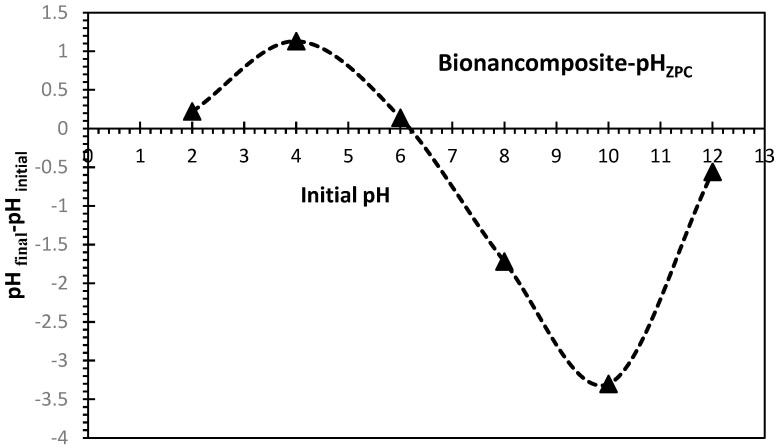
pH at the point of zero charge (pH_ZPC_) of the alginate–clay bio–nanocomposite.

To determine the pH_ZPC_ of the adsorbent, 50 mL of 0.01 M NaCl solution was placed in different 250 mL Erlenmeyer flasks, and 0.5 g of the alginate–clay bio-nanocomposite beads were introduced into each of them. The pH values of these solutions were adjusted between 2 and 12 with 0.1 M HCl/NaOH solutions. These flasks were kept for 48 h, and the final pH of the solutions was measured. The difference between the initial and final pH was measured, and the point where ΔpH = 0 was taken as the point of zero charge (Figure 3). The pH_ZPC_ of the alginate–clay bio-nanocomposite used was found to be 6.2 (Figure 3). This figure shows that the bio-nanocomposite surface under study bears a positive charge at pH levels below the pH_ZPC_, but the surface is dominated by negative charges at pH levels above the pH_ZPC_ [14,33].

#### 3.1.3. Scanning Electron Microscopy (SEM)

**Figure 4:**
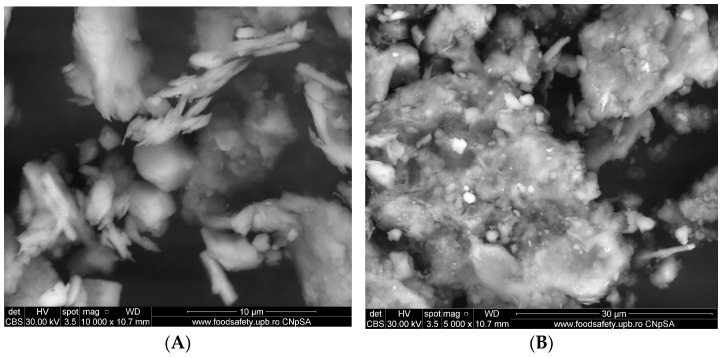

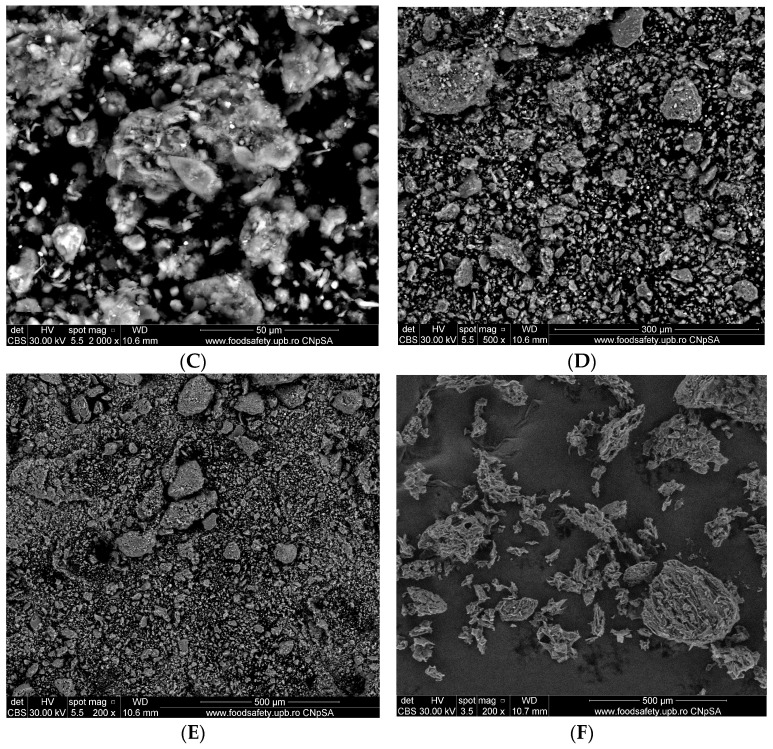
SEM images of clay microparticles (Muscovite) with magnification scale between 10,000 and 200, and scale bar between 10 µm and 500 µm. (**A**) SEM images for clay with magnification scale 10,000 and scale bar 10 µm. (**B**) SEM images for clay with magnification scale 5000 and scale bar 30 µm. (**C**) SEM images for clay with magnification scale 2000 and scale bar 50 µm. (**D**) SEM images for clay with magnification scale 500 and scale bar 300 µm. (**E**) SEM images for clay with magnification scale 200 and scale bar 500 µm. (**F**) SEM images for clay with magnification scale 200 and scale bar 500 µm.

**Figure 5:**
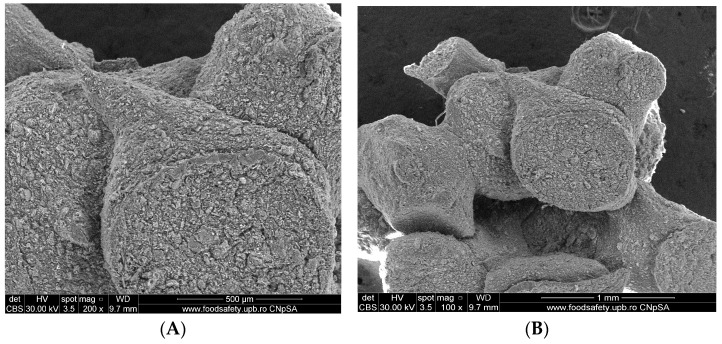

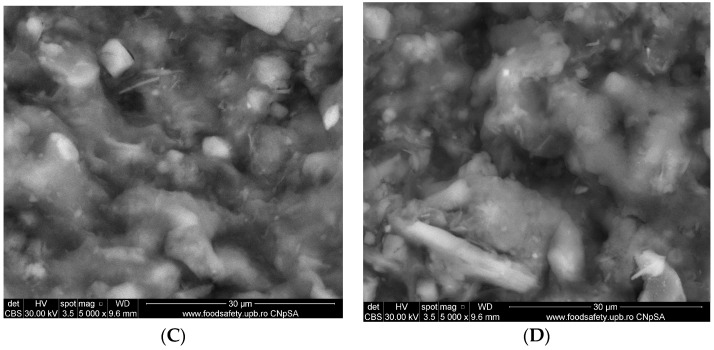
SEM images of alginate–clay bio-nanocomposite microparticles with magnification scale between 5000 and 100, and scale bar between 1 µm and 30 µm. (**A**) SEM images of alginate–clay bio-nanocomposite microparticles with magnification scale 200 and scale bar 500 µm. (**B**) SEM images of alginate–clay bio-nanocomposite microparticles with magnification scale 100 and scale bar 1 µm. (**C**) SEM images of alginate–clay bio-nanocomposite microparticles with magnification scale 5000 and scale bar 30 µm. (**D**) SEM images of alginate–clay bio-nanocomposite microparticles with magnification scale 5000 and scale bar 30 µm.

Scanning electron microscopy enables us to observe grain morphology and, in particular, to estimate their approximate diameter, i.e., the way in which the grains, fibers, and fiber aspects of the processed material are arranged. The alginate–clay bio-nanocomposite was analyzed by SEM coupled with energy-dispersive X-ray analysis (SEM/EDX, Inspect F50 (National University of Science and Technology Politehnica of Bucharest, București, Romania)). The SEM images of the natural clay microparticles (Muscovite) and the alginate–Moroccan clay bio-nanocomposite studied are presented in Figure 4 and Figure 5, respectively.

The images obtained on the clay sample (Figure 4) generally show that this material is characterized by two aspects: the dispersed powder and the formation of a few agglomerates of different shapes, ranging in size from 10 to 50 µm (Images A, B, and C). At higher magnification, these agglomerates are formed by the assembly of small particles of inhomogeneous morphology and different shapes (Images D, E, and F). According to these images, these forms of agglomerates and small particles are characterized by the presence of a large number of pores at the total surface level of the clay used, which will be of interest for use as adsorbents or heterogeneous catalysts.

Figure 5 shows SEM images of alginate–clay bio-nanocomposite microparticles obtained at different magnifications. At high magnification, these images show agglomerates of a very fine powder containing grains of various sizes and inhomogeneous shapes with different dimensions (500 µm and 1 mm) (Images A and B).

Images C and D show that these grains are difficult to individualize, as most of them appear to have coalesced. This shape seems to be very interesting for use in adsorption due to the quantity of cavities and pores observed.

#### 3.1.4. FT-IR Spectroscopy

**Figure 6:**
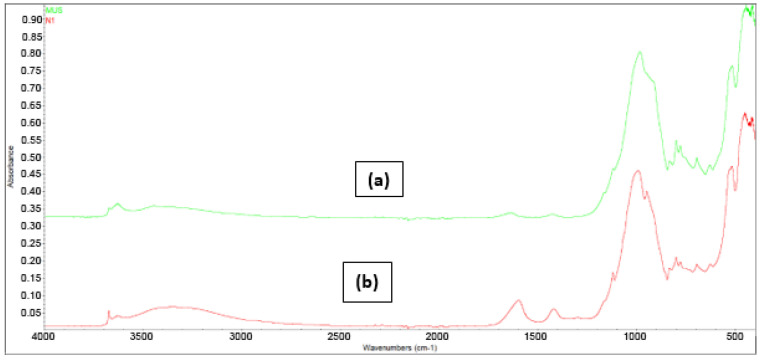
Fourier-transform infrared spectroscopy (FTIR) spectrum: (a) natural clay; (b) alginate–clay bio-nanocomposite.

The IR spectra of natural clay and alginate–clay bio-nanocomposite are shown in Figure 6. FT-IR spectroscopy was employed to investigate the structure and surface functional groups of both Moroccan clay and the alginate–clay bio-nanocomposite. As illustrated in Figure 6, the silica component displays characteristic absorption peaks at specific wavelengths of 470, 520, 640, 700, 780, 800, 950, and 996 cm^−1^. The 470 cm^−1^ band is associated with Si–O stretching vibrations, while the 996 cm^−1^ band is a result of Si–O–Si bending vibrations found in the dioctahedral of the natural clay. Moreover, there are two minor peaks at 520 cm^−1^ [34,35,36], corresponding to Al–O–Si, and at 800 cm^−1^, related to Si–O bending and Si–OH stretching vibrations. The band at 3640 cm^−1^ arises from the stretching vibration of OH units within the clay layers attached to aluminum. In the case of the composite materials, their spectroscopic profiles closely resemble the previously obtained results, featuring distinctive peaks in the 1420 and 1600 cm^−1^ range, which are indicative of COO^−^ vibrations of alginate [37,38].

### 3.2. Factors Controlling the Adsorption of H_2_PO_4_^−^ and HPO_4_^2−^ and NO_3_^−^ Ions from Aqueous Solution by Alginate–Clay Bio-Nanocomposite

#### 3.2.1. Effect of Adsorbent Dosage

The adsorbent dosage is a crucial parameter, as it defines the capacity of an adsorbent for a given initial concentration of the adsorbate [39]. The effect of adsorbent dosage on the removal of orthophosphates (H_2_PO_4_^−^ and HPO_4_^2−^) and nitrates (NO_3_^−^) at *C*_0_ = 100 mg/L was studied by stirring in different masses at 25 °C.

**Figure 7:**
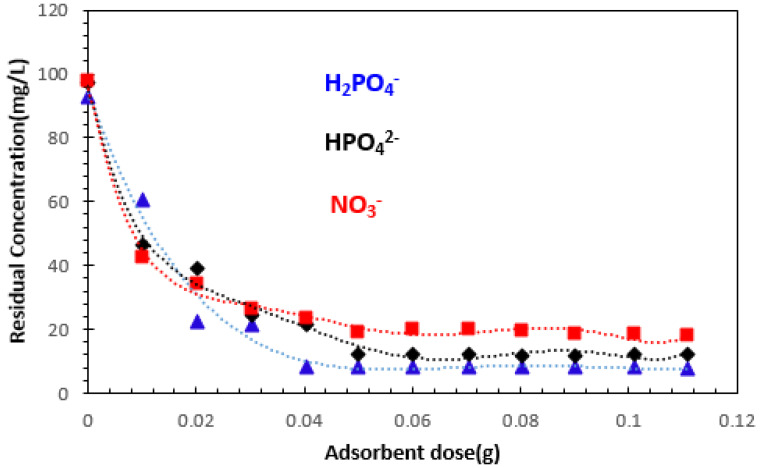
Effect of adsorbent dose on the removal of H_2_PO_4_^−^, HPO_4_^2−^, and NO_3_^−^ ions: *C*_0_ = 100 mg/L, T = 23 ± 2 °C, and Tc = 12 h.

Figure 7 illustrates the variation in the amount of orthophosphate and nitrate ions adsorbed as a function of adsorbent mass.

The removal of H_2_PO_4_^−^, HPO_4_^2−^, and NO_3_^−^ ions increases with the adsorbent dose, which could be explained by the increase in the number of available adsorption sites [15]. From these results, we can see that the relative adsorption capacity expressed in residual concentration decreases with increasing material mass, then stabilizes at an optimum mass equal to 0.06 g for orthophosphate and nitrate ions, where a plateau of maximum adsorption appears.

This may be due to the overlapping of adsorption sites as a result of adsorbent particle clutter. According to several published studies, this behavior can be explained by the fact that a large quantity of adsorbent creates particle agglomerations, resulting in a reduction in the total adsorption surface area and, consequently, a decrease in the quantity of adsorbate per unit mass of adsorbent [14,40,41]. In the following subsections, we will work with an optimum mass of 0.06 g for the ions studied. This result shows that 0.06 g of alginate–clay bio-nanocomposite per 50 mL of solution, corresponding to a mass/volume ratio equal to 1.2 g/L (R = 1.2 g/L), is sufficient to achieve the adsorption equilibrium for H_2_PO_4_^−^, HPO_4_^2−^, and NO_3_^−^ ions, after a contact time of 12 h.

#### 3.2.2. Effect of Contact Time

**Figure 8:**
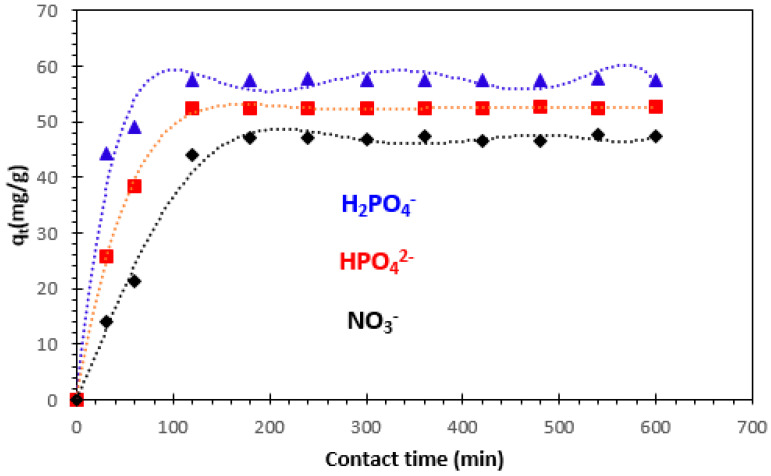
Effect of contact time on the removal of H_2_PO_4_^−^, HPO_4_^2−^, and NO_3_^−^ ions onto bio-nanocomposite beads: m/V = 1.2 g/L and T = 23 °C.

Contact time is considered an interesting operational parameter for an economical wastewater treatment process. The impact of contact time on discontinuous adsorption of H_2_PO_4_^−^, HPO_4_^2−^, and NO_3_^−^ ion solution is illustrated in Figure 8.

From this figure, we can see that the adsorption kinetics of H_2_PO_4_^−^, HPO_4_^2−^, and NO_3_^−^ ions on the nanocomposite used is characterized by two distinct steps, the first relatively rapid and the second indicated by the equilibrium step. The equilibrium time is around 120 min for orthophosphate ions and 180 min for nitrate ions.

These times are more than sufficient to establish equilibrium for the study of parameters which influence the removal of H_2_PO_4_^−^, HPO_4_^2−^, and NO_3_^−^ ions by the alginate–clay bio-nanocomposite studied. When equilibrium is established, the adsorption rate stabilizes. The increase in the amount adsorbed as a function of time in the first stage may be due to the large number of vacant sites available on the adsorbent surface [40].

#### 3.2.3. Effect of Initial Solution pH

The pH can have an impact on the structure of both adsorbent and adsorbate, as well as on the adsorption mechanism. This is due to the existence of protons that can modify the charge on the surface of adsorbent [41,42].

**Figure 9:**
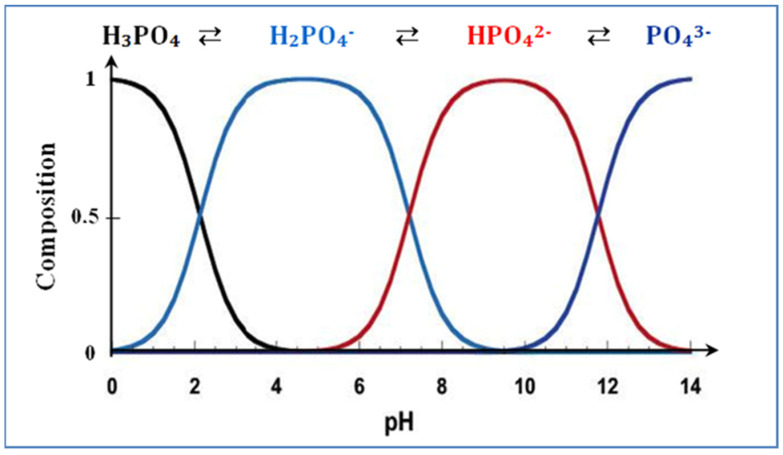
Effect of pH on the distribution of orthophosphate ions in solution.

The pH plays a critical role in determining the stability and the prevailing forms of orthophosphate ions, including H_2_PO_4_^−^, HPO_4_^2−^, and PO_4_^3−^ [43]. The main phosphate ions in acidic solution are H_2_PO_4_^−^ and HPO_4_^2−^, while under alkaline conditions, they exist as PO_4_^3−^. Figure 9 shows the relative predominance of the different forms of orthophosphate ions as a function of pH, under standard conditions. Phosphoric acid is a triacid and dissociates in the following sequence [10,44,45].

H_3_PO_4_ + H_2_O ⇄ H_2_PO_4_^−^ + H_3_O^+^ (pKa_1_ = 2.1)

H_2_PO_4_^−^ + H_2_O ⇄ HPO_4_^2−^ + H_3_O^+^ (pKa_2_ = 7.2)

HPO_4_^2−^ + H_2_O ⇄ PO4_3_^−^ + H_3_O^+^ (pKa_3_ = 12.4)

**Figure 10:**
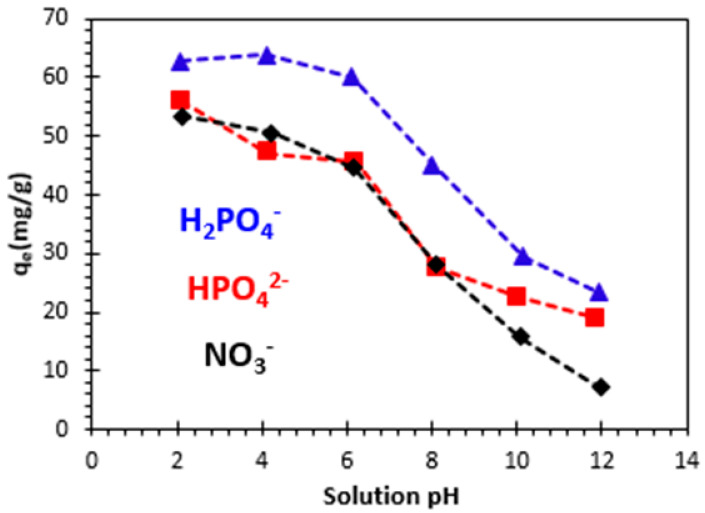
Effect of initial pH on adsorption of H_2_PO_4_^−^, HPO_4_^2−^, and NO_3_^−^ ions: *C*_0_ = 100 mg/L, m/V = 1.2 g/L, and T = 23 ± 2 °C.

The examination of the pH impact was carried out according to the following method. In the initial step, 0.06 g of bio-nanocomposite beads were introduced into multiple flasks, each containing 50 milliliters of H_2_PO_4_^−^, HPO_4_^2−^, and NO_3_^−^ ion solution. The study of the effect of solution pH on the adsorption of phosphate and nitrate ions was carried out at an initial concentration of 100 mg/L and at different pH. The pH ranged from 2.08 to 11.95 for H_2_PO_4_^−^, from 2.12 to 11.90 for HPO_4_^2−^, and from 2.10 to 11.96 for NO_3_^−^. The results are shown in Figure 10. This figure shows that the adsorbed quantities of orthophosphate and nitrate ions remain significantly higher, at pH values below the pH_ZPC_ of the bio-nanocomposite. These results show that the adsorbed amount of H_2_PO_4_^−^, HPO_4_^2−^, and NO_3_^−^ ions is slightly increased, when the initial pH of the solution is below six, and then decreased in the basic medium, which can be explained by the pH value of the zero-charge point. We can see that, at pH values below the pH of the zero point charge (pH < pH_ZPC_), the removed amount of orthophosphate and nitrate ions is significant. The surface of the bio-nanocomposite used at pH< pH_ZPC_ is positively charged, which favors anion adsorption. This can be explained by the electrostatic attraction between the anions and the positively charged adsorbent surface. At pH values above pH_ZPC_, due to the presence of hydroxide ions (OH^−^), the surface of the bio-nanocomposite is negatively charged, leading to a reduction in the quantity adsorbed. For the adsorption of orthophosphate and nitrate ions, the surface of the bio-nanocomposite must be positively charged [46,47]. The decrease in adsorption capacity when pH increases can be explained by the fact that, at a higher pH, there are more OH^−^ ions in the solution that are likely to compete with orthophosphates, resulting in a noticeable decrease in adsorption towards the studied anions. Additionally, the surface of the adsorbent becomes more negatively charged at a higher pH, leading to greater repulsion, and thus a decrease in phosphate removal [3].

A similar study by Elemile et al. (2022) [48] on the removal of nitrate ions by modified chicken feathers (MCFs) showed that the highest nitrate removal efficiency was observed in the initial pH range below eight. When the initial pH is less than eight, the adsorption removal efficiency of nitrates increases along with the pH increase, as shown in Figure 10, and begins to decrease when the initial pH value is greater. For pH values below 8.0, the decrease in removal efficiency could be caused by dissociation of functional groups on the adsorbent.

#### 3.2.4. Effect of Temperature

**Figure 11:**
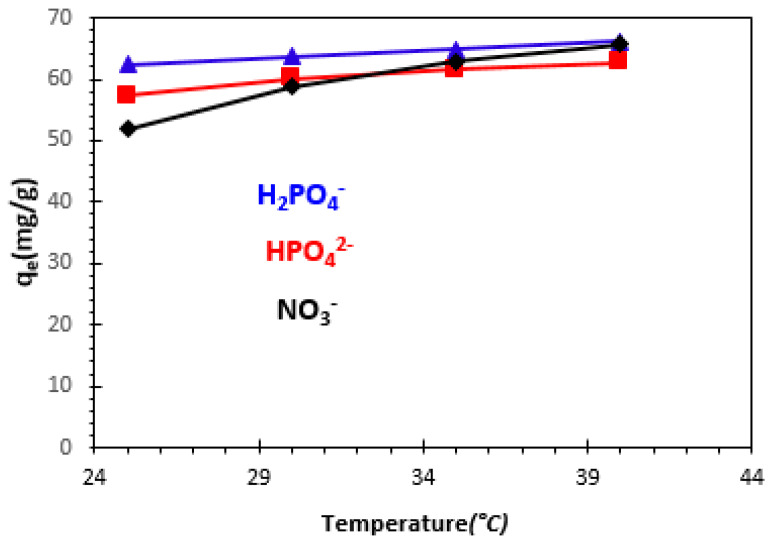
Effect of temperature on adsorption of H_2_PO_4_^−^, HPO_4_^2−^, and NO_3_^−^ ions by bio-nanocomposite beads: C_0_ = 100 mg/L and m/V = 1.2 g/L.

The influence of solution temperature on the adsorption of H_2_PO_4_^−^, HPO_4_^2−^, and NO_3_^−^ ions was tested over a temperature range from 25 to 40 °C, with an initial concentration of 100 mg/L (Figure 11). Increasing the temperature from 25 °C to 40 °C resulted in a slight increase in the adsorption capacity of the prepared bio-nanocomposite. These results lead to the conclusion that adsorption capacity increases with increasing temperature, suggesting endothermic adsorption. These results can be verified by determining the thermodynamic parameters.

A similar study by Morghi et al. (2015) [47] on nitrate ion adsorption using chitin showed that the concentration of NO_3_^−^ adsorbed by chitin increases slightly with temperature, and that this adsorption would be endothermic. The optimum temperature at equilibrium contact time for the adsorption of nitrate ions onto chitin was obtained at 35 °C. Abidar et al. (2015) [49] studied the evolution of the retained concentration of H_2_PO_4_^−^ and HPO_4_^2−^ ions as a function of temperature for an initial concentration of 100 mg/L. This study showed that the concentration of retained orthophosphate ions (*Cr*) increases with increasing temperature.

### 3.3. Adsorption Kinetic Models

The experimental data on adsorption kinetics were examined using a variety of kinetic models, namely the pseudo-first-order model, pseudo-second-order model, Elovich model, and intra-particle diffusion model [13].

#### 3.3.1. Pseudo-First-Order Kinetics Model

The kinetics equation of the pseudo-first-order model and its linearized form may be represented as follows [13,50,51,52,53,54]:

(3) $$\frac{dq_{t}}{dt}=k_{1}\left(q_{e}-q_{t}\right)\;\;\;\;\left(non-linear\;form\right)\;\;\;$$

(4) $$\ln\left(q_{e}-q_{t}\right)=\ln q_{e}-k_{1}t\;\;\;\;\;\left(linear\;form\right)\;\;$$

where *k*_1_ (min^−1^) is the rate constant for the pseudo-first-order kinetics model, *q_e_* (mg.g^−1^) and *q_t_* (mg.g^−1^) are the amounts of H_2_PO_4_^−^, HPO_4_^2−^, and NO_3_^−^ ions retained on weight unit of adsorbent at equilibrium, and at any time *t* (min), respectively.

**Figure 12:**
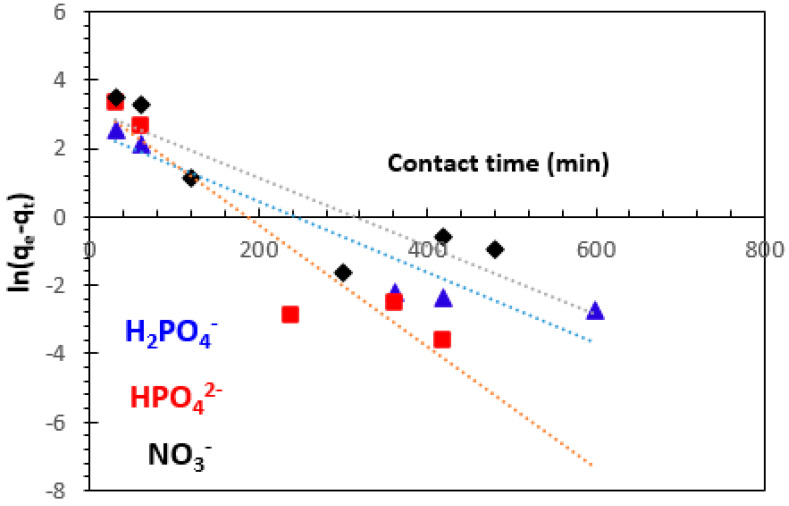
Pseudo-first-order adsorption kinetics on adsorption of H_2_PO_4_^−^, HPO_4_^2−^, and NO_3_^−^ ions by bio-nanocomposite beads.

The plot of *ln (q_e_−q_t_)* versus contact time t for alginate–clay bio-nanocomposite gives a straight line of slope –*k*_1_ and intercepts *ln q_e_* (Figure 12). The values of the theoretical adsorption capacity *(q_e_*_,the_), the rate constant for the pseudo-first-order kinetics model (*k*_1_), and the correlation coefficient (R^2^) are presented in Table 1. The table shows that the value of theoretical adsorbed amount *q_e_* is not quite similar to the experimental value (*q_e,_*_the_ = 6.853 mg/g < *q_e_*_,exp_ = 62.37 mg/g for H_2_PO_4_^−^, *q_e_*_,the_ = 9.079 mg/g < *q_e_*_,exp_ = 57.30 mg/g for HPO_4_^2−^, and *q_e,_*_the_ = 8.584 mg/g < *q_e,_*_exp_ = 51.85 mg/g for NO_3_^−^), suggesting the insufficiency of pseudo-first-order model. We find that, under these conditions, the pseudo-first-order model is not adequate to describe the adsorption kinetics of H_2_PO_4_^−^, HPO_4_^2−^, and NO_3_^−^ from aqueous solutions onto bio-nanocomposite beads.

#### 3.3.2. Pseudo-Second-Order Kinetics Model

**Figure 13:**
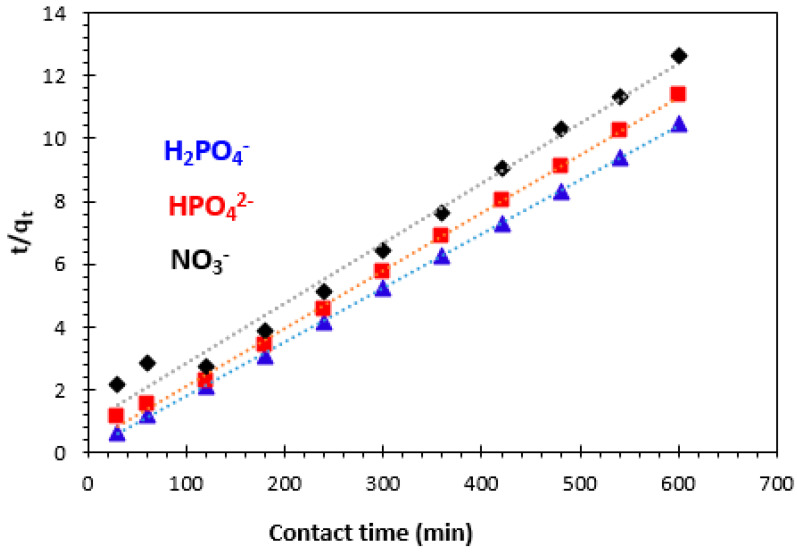
Pseudo-second-order kinetic model of H_2_PO_4_^−^, HPO4^2−^, and NO_3_^−^ ion adsorption.

The rate equation and its linearized form for the pseudo-second-order kinetics model can be represented as follows [13,50,51,52,53,54]:

(5) $$\frac{dq_{t}}{dt}=k_{2}{\left(q_{e}-q_{t}\right)}^{2}\;\;\left(non-linear\;form\right)$$

(6) $$\frac{t}{q_{t}}=\frac{1}{k_{2}q_{e}^{2}}+\left(\frac{1}{q_{e}}\right)t\;\;\;\;\;\left(linear\;form\right)\;\;$$

where *k*_2_ (g.mg^−1^min^−1^) is the rate constant for the pseudo-second-order kinetics model, *q_e_* (mg/g) and *q_t_* (mg/g) are the amounts of H_2_PO_4_^−^, HPO_4_^2−^, and NO_3_^−^ ions retained on weight unit of adsorbent at equilibrium, and at any contact time *t* (min), respectively. The pseudo-second-order plots of H_2_PO_4_^−^, HPO_4_^2−^, and NO_3_^−^ adsorption are presented in Figure 13, and the kinetic parameters are given in Table 1. The correlation coefficient for the pseudo-second-order kinetic model is nearly equal to one (>0.99), and the value of theoretical adsorption capacity (*q_e_*_,the_) is comparable to the experimental one (*q_e_*_,the_ = 58.14 mg/g for H_2_PO_4_^−^, *q_e_*_,the_ = 54.64 mg/g for HPO_4_^2−^, and *q_e_*_,the_ = 52. 63 mg/g for NO_3_^−^).

Therefore, it was concluded that the pseudo-second-order adsorption model is more appropriate to describe the adsorption kinetics of H_2_PO_4_^−^, HPO_4_^2−^, and NO_3_^−^ ions on bio-nanocomposite.

#### 3.3.3. Elovich Kinetic Model

The Elovich model is applicable to systems with heterogeneous surfaces and is particularly suitable for describing chemisorption kinetics [13]. The model can be expressed using Equations (7) and (8), where the terms *q_e_* (mg/g) and *q_t_* (mg/g) represent the amounts of H_2_PO_4_^−^, HPO_4_^2−^, and NO_3_^−^ adsorbed at equilibrium and at any given contact time *t* (minutes), respectively.

The Elovich model is valid for systems with heterogeneous surface and is suitable for chemisorption kinetics. The equation for Elovich kinetic model and its linearized form may be expressed as:

(7) $$\frac{dq_{t}}{dt}=\alpha e^{-\beta q_{t}}\;\;\;\left(non-linear\;form\right)$$

**Figure 14:**
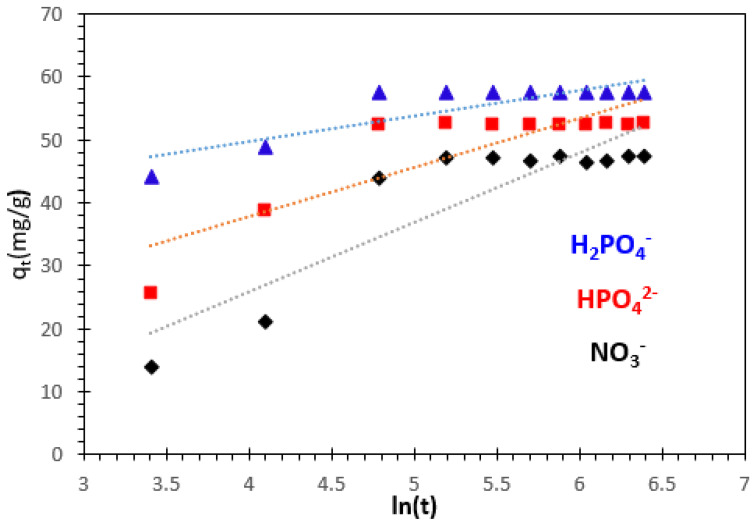
Elovich kinetic model of H_2_PO_4_^−^, HPO4^2−^, and NO_3_^−^ ion adsorption.

(8) qt=ln⁡(αβ)β+1β ln⁡(t)   linear form

where *q_e_* (mg.g^−1^) and *q_t_* (mg.g^−1^) are the amounts of H_2_PO_4_^−^, HPO_4_^2−^, and NO_3_^−^ adsorbed at equilibrium, and at any contact time *t* (min), respectively. *α* (mg.g^−1^.min^−1^) is the initial adsorption rate, and *β* (g.mg^−1^) is the desorption constant related to the extent of the surface coverage and activation energy for chemisorption. The Elovich kinetic constants *α* and *β* are obtained from the intercept and the slope, respectively (Figure 14). The correlation coefficient indicates that the Elovich model is not adequate to characterize the H_2_PO_4_^−^, HPO_4_^2−^, and NO_3_^−^ ion adsorption on nanocomposite beads.

#### 3.3.4. Intra-Particle Diffusion Kinetics Model

**Figure 15:**
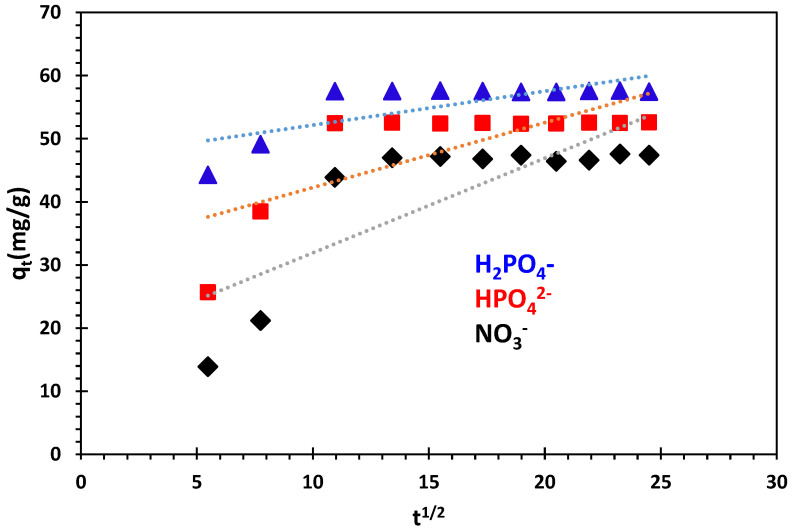
Intra-particle diffusion kinetics model of H_2_PO_4_^−^, HPO_4_^2−^, and NO_3_^−^ ion adsorption.

The intra-particle diffusion model is of significant interest because internal diffusion determines the adsorption rate in most liquid systems [13]. To calculate the initial rate of intra-particle diffusion, Equation (9) is linearized. In this equation, *k_p_* (mg^−1^·min^1/2^) represents the intra-particle diffusion rate constant; *c* (mg·g^−1^) is the concentration of H_2_PO_4_^−^, HPO_4_^2−^, and NO_3_^−^ ions from the solution at equilibrium; and *q_t_* (mg·g^−1^) denotes the amount of orthophosphate and nitrate ions retained on a unit weight of the adsorbent, at contact time *t* (minutes). The graphical representation of this relationship is given in Figure 15.

(9) $$q_{t}=k_{p}t^{1/2}+c\;\;\;\;\left(linear\;form\right)$$

where *k_p_* (mg·g^−1^· min^1/2^) is the intra-particle diffusion rate constant; *c* (mg·g^−1^) is the concentration of H_2_PO_4_^−^, HPO_4_^2−^, and NO_3_^−^ ions from solution at equilibrium; and *q_t_* (mg·g^−1^) is the amount of H_2_PO_4_^−^, HPO_4_^2−^, and NO_3_^−^ ions retained on weight unit of adsorbent, at contact time *t* (min). The values of intra-particle diffusion, *k_p_*, were obtained from the slope of the straight-line portions of plot of *q_t_* versus *t*^1/2^ for various temperatures of the solution. The correlation coefficients (R^2^) for the three anions studied are 0.568 for H_2_PO_4_^−^, 0.554 for HPO_4_^2−^, and 0.630 for NO_3_^−^ at 25 °C. This correlation coefficient indicates that the intra-particle diffusion model is not suitable to describe the kinetics of H_2_PO_4_^−,^ HPO_4_^2−^, and NO_3_^−^ ion adsorption from aqueous solutions on bio-nanocomposite beads. The values of *k_p_* and *c* calculated from the slopes and intercepts are summarized in Table 1.

The adsorption study of H_2_PO_4_^−^, HPO_4_^2−^, and NO_3_^−^ ions by the bio-nanocomposite showed that the correlation coefficient (R^2^) values for the pseudo-second-order adsorption kinetic model were significantly high (around 0.99) for an initial concentration equal to 100 mg/L. Furthermore, the adsorption capacity calculated using the pseudo-second-order model closely matched the results found experimentally. It can be deduced that the pseudo-second-order adsorption model is considered the most appropriate choice for elucidating the kinetics of H_2_PO_4_^−^, HPO_4_^2−^, and NO_3_^−^ ion adsorption by the nanocomposites used.

### 3.4. Isotherm Study

Adsorption isotherms are mathematical models that describe the distribution of adsorbed species between the solid and liquid phases and are significant data for studying the adsorption mechanism [55]. In this study, the Langmuir, Freundlich, Temkin, and Dubinin–Radushkevich (D–R) equations were used to describe the relationship between the adsorption of H_2_PO_4_^−^, HPO_4_^2−^, and NO_3_^−^ ions on alginate–clay bio-nanocomposite. This study was performed by ranging the initial ion concentration from 100 to 600 mg/L at room temperature.

#### 3.4.1. Langmuir Adsorption Isotherm

The Langmuir adsorption isotherm supposes that the solid surface has a finite number of identical sites, which shows homogeneous surfaces [13,55,56,57,58]. The Langmuir equation and its linearized form may be represented as follows:

(10) $$q_{e}=q_{L}\frac{K_{L}C_{e}}{1+K_{L}C_{e}}\;\;\;(non-linear\;form)\;$$

(11) $$\frac{1}{q_{e}}=\frac{1}{q_{L}}+\frac{1}{q_{L}K_{L}C_{e}}\;\;\;\;\;\;(linear\;form)\;\;\;$$

where *q_e_* (mg/g) is the amount adsorbed at equilibrium concentration *C_e_* (mg/L), *q_L_* (mg/g) is the Langmuir constant representing maximum monolayer capacity, and *K_L_* (L/mg) is the Langmuir constant related to the energy of adsorption.

**Figure 16:**
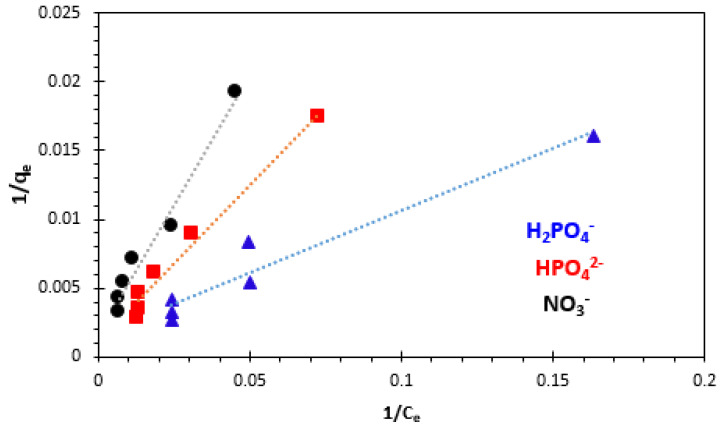
Langmuir adsorption isotherm of H_2_PO_4_^−^, HPO_4_^2−^, and NO_3_^−^ ions at 25 °C.

The plots of 1/*q_e_*function of 1/*C_e_* for the adsorption of H_2_PO_4_^−^, HPO_4_^2−^, and NO_3_^−^ ions are given in Figure 16. The values of the adsorption capacity (*q_L_*), the Langmuir constant (*K_L_*), and the correlation coefficient (R^2^) are presented in Table 3.

The Langmuir model is an indication of surface homogeneity of the adsorbent. The basic assumption of Langmuir adsorption isotherm is also based on monolayer coverage of the adsorbate on the surface of the adsorbent. The adsorption capacity of the adsorbent decreased as the temperature increased. The highest value of *q_L_* obtained at 25 °C was 625 mg/g for H_2_PO_4_^−^, 909.09 mg/g for HPO_4_^2−^, and 588.23 mg/g for NO_3_^−^ ions (Table 3).

The essential feature of the Langmuir isotherm can be expressed by means of ‘*R_L_’*, a dimensionless constant referred to as a separation factor or equilibrium parameter to predict whether an adsorption system is favorable or unfavorable. *R_L_* is calculated using Equation (12).

(12) $$R_{L}=\frac{1}{1+K_{L}C_{0}}$$

where *K_L_* (L.mol^−1^) is the Langmuir constant, and *C*_0_ (mol.L^−1^) is the highest initial ion concentration.

The calculated values of parameter *R_L_* for this study were found to be between 0 and 1 (0.495 for H_2_PO_4_^−^, 0.261 for HPO_4_^2−^, and 0.270 for NO_3_^−^), indicating that the adsorption of H_2_PO_4_^−^, HPO_4_^2−^, and NO_3_^−^ ions onto bio-nanocomposite bead particles was favorable (Table 2).

#### 3.4.2. Freundlich Adsorption Isotherm

**Figure 17:**
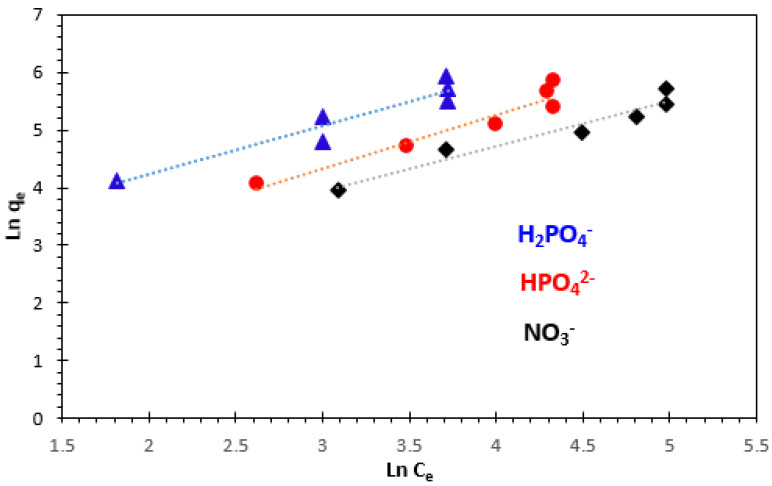
Freundlich adsorption isotherm of H_2_PO_4_^−^, HPO_4_^2−^, and NO_3_^−^ ions at 25 °C.

The Freundlich equation provides the most suitable adsorption data for heterogeneous natural adsorbents. The Freundlich adsorption isotherm equation and its linear form can be written as follows [13,55,56,57]:

(13) $$q_{e}=K_{F}{C_{e}}^{1/n}\;\;\;\;\;\;\;\;\;\;\;\left(non-linear\;form\right)$$

(14) $$\ln q_{e}=\ln K_{F}+\frac{1}{n}\ln C_{e}\;\;\;\;\;\;\;\left(linear\;form\right)$$

where *q_e_* (mg/g) is the amount of H_2_PO_4_^−^, HPO_4_^2−^, and NO_3_^−^ ions adsorbed per unit weight of adsorbent; *C_e_* (mg/L) is the equilibrium concentration of solute in the bulk solution; *K_F_* (mg/g) is the Freundlich constant, which is a comparative measure of the adsorption capacity of the adsorbent; and *n* is an empirical constant related to heterogeneity of the adsorbent surface. The parameter *n* also indicates the nature of the adsorption process. The value of *n* lies between 0 and 1 for a favorable adsorption, while *n* > 1 represents an unfavorable adsorption, and *n* = 1 represents the linear adsorption, while the adsorption operation is irreversible if *n* = 0. The isotherm constants *n* and *K_F_* were calculated from the slope and intercept of the plot *ln q_e_* versus *lnC_e_* (Figure 17). The values for Freundlich constants and correlation coefficients (R^2^) for both temperatures are also presented in Table 3.

The Freundlich isotherm constants *K_F_* and *n* are constants incorporating all factors which influence the adsorption process such as adsorption capacity and intensity of adsorption. The constants *K_F_* and *n* were calculated from Equation (12). These experiments confirm the efficiency of the bio-nanocomposite used to remove H_2_PO_4_^−^, HPO_4_^2−^, and NO_3_^−^ ions from aqueous solutions.

#### 3.4.3. Temkin Isotherm

**Figure 18:**
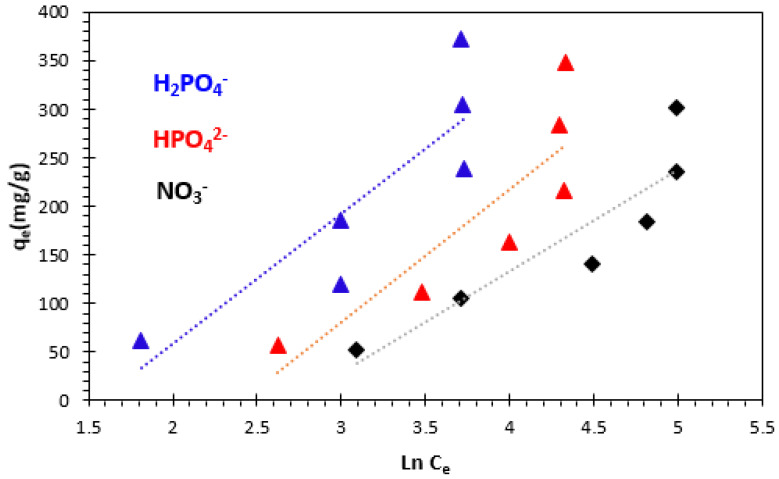
Temkin adsorption isotherm of H_2_PO_4_^−^, HPO_4_^2−^, and NO_3_^−^ ions at 25 °C.

The Temkin adsorption isotherm model is based on the heat of ion adsorption, which is due to the interactions between the adsorbate and the adsorbent. The Temkin isotherm equation is given as follows [13,55]:

(15) $$q_{e}=\frac{RT}{b_{T}}\ln K_{T}C_{e}\;\;\left(non-linear\;form\right)$$

(16) $$q_{e}=\frac{RT}{b_{T}}\ln K_{T}+\frac{RT}{b_{T}}\ln C_{e}\;\left(linear\;form\right)$$

where *T* is absolute temperature in Kelvin, and *R* the universal gas constant (8.314 J.K^−1^.mol^−1^); *b_T_* (J.mol^−1^) is the Temkin isotherm constant related to the heat of adsorption; *K_T_* (L.mg^−1^) is the equilibrium binding constant corresponding to the maximum binding energy. The Temkin isotherm plot is presented in Figure 18, and the isotherm parameters are given in Table 3. The Temkin constants *b_T_* related to heat of adsorption of H_2_PO_4_^−^, HPO_4_^2−^, and NO_3_^−^ ions at 25 °C were found to be 18.509 J.mol^−1^ for H_2_PO_4_^−^, 15.92 J.mol^−1^ for HPO_4_^2−^, and 23.73 J.mol^−1^ for NO_3_^−^, respectively, at 25 °C (Table 3).

Linear regression of the data points showed rather low R^2^ values ranging from 0.757 to 0.813, indicating that the adsorption of H_2_PO_4_^−^, HPO_4_^2−^, and NO_3_^−^ ions did not fully follow the Temkin isotherm.

#### 3.4.4. Dubinin–Radushkevich (D–R) Isotherm

Dubinin–Radushkevich isotherm is commonly used to express the adsorption mechanism with a Gaussian energy distribution onto a heterogeneous surface. It is not based on the assumption of a homogeneous surface or constant adsorption potential, but it is applied to estimate the mean free energy of adsorption (*E*). The non-linear and linear forms of the D–R equation can be written as follows [13]:

(17) $$q_{e}=q_{m}e^{{-K}_{p}\varepsilon^{2}}\;\;\left(non-linear\;form\right)$$

(18) $$\ln q_{e}=\ln q_{m}{-K}_{D}\varepsilon^{2}\;\;\left(linear\;form\right)\;\;\;\;\;\;$$

where *q_m_* (mg/g) is the theoretical saturation capacity, and *ε* is the Polanyi potential that can be calculated from Equation (19):

(19) $$\varepsilon =RT\ln\left(1+\frac{1}{C_{e}}\right)$$

The constant *K_D_* (mol^2^/J^2^) gives an idea about the mean free energy *E* (kJ/mol) of adsorption per molecule of the adsorbate, when it is transferred to the surface of the solid from the bulk solution, and can be calculated from the *K_D_* value using the following relation (Equation (20)):

(20) $$E=\frac{1}{{\left(2K_{D}\right)}^{1/2}}$$

**Figure 19:**
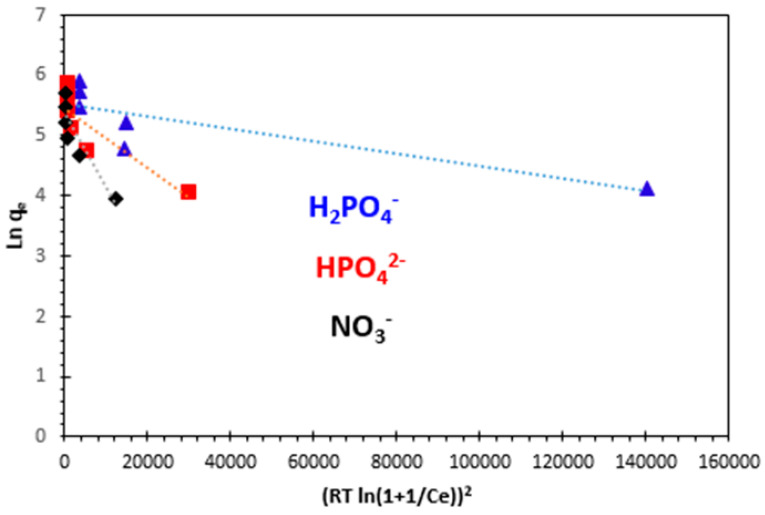
Dubinin−Radushkevich (D–R) adsorption isotherm of H_2_PO_4_^−^, HPO_4_^2−^, and NO_3_^−^ ions at 25 °C.

This parameter gives information on the adsorption mechanism if it is a chemical ion exchange or a physical adsorption. If the value of *E* is between 8 and 16 kJ/mol, the adsorption process is expected to be chemisorption, while for values of *E* < 8 kJ/mol, the adsorption process is physical in nature. The results are illustrated in Table 3. The slope of the plot of *ln q_e_* versus *ε^2^* gives *K_D_*, and the intercept yields the adsorption capacity *q_m_*. As can be seen in Figure 19 and Table 3, the correlation coefficient values are 0.717 for H_2_PO_4_^−^, 0.747 for HPO_4_^2−^, and 0.821 for NO_3_^−^, respectively, at 25 °C. The numerical value of adsorption of the mean free energy is of 223.60 J.mol^−1^ for H_2_PO_4_^−^, 44.72 J.mol^−1^ for HPO_4_^2−^, and 70.71 J.mol^−1^ for NO_3_^−^ (Table 3), corresponding to a physisorption and the predominance of van der Waals forces.

### 3.5. Thermodynamic Study

The correlation between temperature and adsorption primarily relies on the specific adsorbent and adsorbate combination, making the determination of thermodynamic parameters crucial for understanding this relationship. In general, adsorption is always accompanied by a thermal effect, which can be either exothermic (Δ*H*° < 0) or endothermic (Δ*H*° > 0). The measurement of the heat change (Δ*H*°*)* serves as the primary criterion to distinguish between chemisorption and physisorption. Furthermore, assessing the standard Gibbs free energy change (Δ*G*°) allows us to predict the spontaneity of a process, while the standard entropy change (Δ*S*°) helps gauge the level of disorder within the adsorbate–adsorbent system [13,14,59,60].

These thermodynamic parameters were calculated from the following equations:

(21) $$\Delta G{}^{\circ}=-RTln\left(K_{d}\right)$$

(22) $$ln\left(Kd\right)=-\frac{\Delta H{}^{\circ}}{RT}+\frac{\Delta S{}^{\circ}}{R}$$

(23) ΔG° =ΔH°−TΔS°

where *T* is the absolute temperature in Kelvin, and *R* is the universal gas constant (8.314 J/mol/K); *K_d_* (L/mol) is the distribution coefficient. Table 4 presents the results for the thermodynamic parameters. The endothermic and physical nature of the adsorption is suggested by the positive values of Δ*H*°, which is consistent with experimental findings. The adsorption process is spontaneous, as evidenced by the negative Δ*G*° values. Positive Δ*S*° values indicate increased randomness at the solid/liquid interface during adsorption of ion-organic ions onto the engineered bio-nanocomposite.

### 3.6. Adsorption Mechanisms

**Figure 20:**
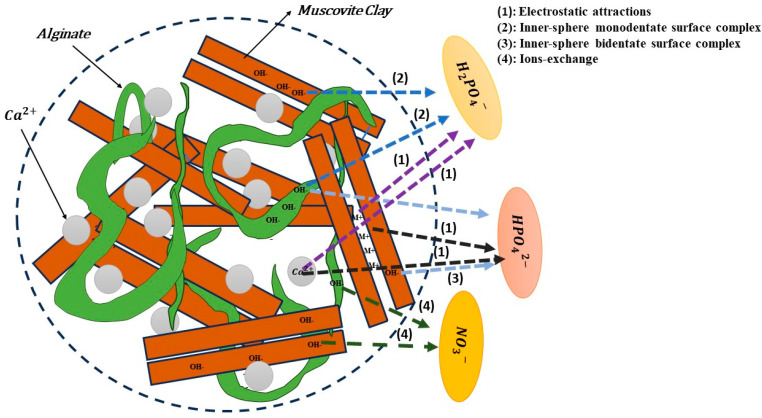
Adsorption mechanisms of for H_2_PO_4_**^−^**, HPO_4_^2−^, and NO_3_^−^ ions on alginate–clay bio-nanocomposite.

To enhance the production processes and optimize the practical applications of the bio-composite, it is essential to delve into the mechanisms of H_2_PO_4_^−^, HPO_4_^2−^, and NO_3_^−^ ion adsorption. The majority of examined bio-composites belong to this category. As illustrated in Figure 20, the essential mechanisms involve electrostatic attractions, monodentate surface complexation within the inner-sphere, bidentate surface complexation within the inner-sphere, and ion exchange. These mechanisms are pivotal for understanding the adsorption of phosphate onto bio-composites [61]. Electrostatic attraction is almost always a necessary step between the anionic functions of negatively charged H_2_PO_4_^−^, HPO_4_^2−^, and NO_3_^−^ ions and the protonated sites of the bio-nanocomposite surface. This observation is logical because cations contribute specifically Ca^2+^ and Al^3+^ [31]. The existence of inner-sphere surface complexes (M–O–P) indicates an interaction between phosphate ions and the bio-nanocomposite. This inference is drawn from observing that the surface hydroxyl groups (M–OH) of bio-nanocomposite could potentially undergo exchange with the adsorbed phosphate ions [62]. As the pH decreases below 7.5 (Figure 9), it is expected that surface complexes undergo protonation, causing a further asymmetry reduction in the phosphate adsorption complexes; at pH < 7.5, the complexes are likely to be protonated, bidentate binuclear complexes. There is a transformation of non-protonated bidentate binuclear complexes at pH > 6 into monoprotonated bidentate binuclear surface complexes as the pH decreases from 4 to 6, as suggested by Yuji and Sparks (2001) [63]. Exchangeable anions, such as chloride ions and OH^−^ ions, can adsorb nitrate through electrostatic forces of attraction, subsequently leading to ion exchange among exchangeable anions. Quaternized sites in the adsorbent frequently play a role in the anion-exchange mechanism, as illustrated in Figure 20. Additionally, nitrate can readily exchange with the interlayer OH^−^ ion of metal hydroxide [64].

### 3.7. Comparison with Published Data

In order to situate our adsorbent among those used to remove inorganic pollutants from aqueous solutions, the maximum experimental adsorption capacity of alginate–Moroccan clay bio-nanocomposite adsorbent was compared to the maximum uptakes (*q_m_*, mg/g) of other adsorbents reported in the literature.

The adsorption of inorganic pollutants on different adsorbents reported in the literature is presented in Table 5. The *q_m_* of this study was found to be comparable with those of other adsorbents. The results indicated that alginate–Moroccan clay adsorbent has a great potential to be used in the treatment of inorganic-pollutant-contaminated wastewater.

## 4. Conclusions

This research delved into the equilibrium and adsorption dynamics of H_2_PO_4_^−^, HPO_4_^2−^, and NO_3_^−^ ions from aqueous solutions, employing the batch equilibration technique. The adsorption process was found to be significantly influenced by some environmental factors such as solution pH, contact time, and temperature. To study the kinetics of H_2_PO_4_^−^, HPO_4_^2−^, and NO_3_^−^ ion adsorption on bio-composite beads, four kinetic models were applied. The pseudo-second-order model exhibited the most accurate correlation with the data in all cases, and the experimental values of *q_e_*_,exp_ matched well with the theorical values. The pseudo-second-order kinetic model showed an excellent fit to the adsorption behavior of H_2_PO_4_^−^, HPO_4_^2−^, and NO_3_^−^ ions on bio-nanocomposite beads at various initial ion concentrations. For the mathematical description of the adsorption equilibrium, the Langmuir, Freundlich, Temkin, and Dubinin–Radushkevich (D–R) adsorption models were studied. Among these models, the Langmuir adsorption isotherm best described the experimental data. The endothermic and physical nature of the adsorption is suggested by the positive values of ΔH°, which is consistent with experimental findings. The adsorption process is spontaneous, as evidenced by the negative ΔG° values. The positive ΔS° values indicate increased randomness at the solid/liquid interface during adsorption of ion-organic ions onto the engineered bio-nanocomposite. The obtained results showed that alginate-based bio-nanocomposites have a highly significant adsorption ability for the removal of H_2_PO_4_^−^, HPO_4_^2−^, and NO_3_^−^ ions in aqueous settings, which is scientifically verified.

## Figures and Tables

**Table 1:** Parameters of four kinetic models for H_2_PO_4_^−^, HPO_4_^2−^, and NO_3_^−^ ion adsorption.

Kinetics Model	Parameters	Phosphate Ions		Nitrate Ions
		H_2_PO_4_^−^	HPO_4_^2−^	NO_3_^−^
Pseudo-first-order model	*q_e_*_,exp_ (mg.g^−1^)	62.37	57.30	51.85
	*K*_1_ (min^−1^)	0.0104	0.0178	0.0101
	*q_e_*_,cal_ (mg.g^−1^)	6.853	9.079	8.584
	R^2^	0.916	0.888	0.771
Pseudo-second-order model	*q_e_*_,exp_ (mg.g^−1^)	62.37	57.30	51.85
	*K*_2_ (g.mg^−1^min^−1^)	0.0030	0.0011	3.7 × 10^−4^
	*q_e_*_,the_ (mg.g^−1^)	58.139	54.64	52.63
	R^2^	0.999	0.998	0.986
Elovich model	*q_e_*_,exp_ (mg.g^−1^)	62.37	57.30	51.85
	*α*	91.46	18.05	50.06
	*β*	0.246	0.128	0.090
	R^2^	0.746	0.737	0.794
Intra-particle diffusion	*K_Int_* (mg.g^−1^min^−1/2^)	0.537	1.027	1.488
	*C_I_*	46.78	31.99	16.94
	R^2^	0.568	0.554	0.630

**Table 2:** Isotherm type for various *R_L_* values.

Ions	Orthophosphates		Nitrates
	H_2_PO_4_^−^	HPO_4_^2−^	NO_3_^−^
*R_L_*	0.495	0.261	0.270

**Table 3:** Parameters of isotherm models for H_2_PO_4_^−^, HPO_4_^2−^, and NO_3_^−^ ion adsorption.

Model	Parameters	Orthophosphate Ions		Nitrate Ions
		H_2_PO_4_^−^	HPO_4_^2−^	NO_3_^−^
Langmuir	*q_L_* (mg/g)	625	909.09	588.23
	*K_L_* (L/mg)	0.017	0.0047	0.0045
	R^2^	0.940	0.975	0.970
Freundlich	1/*n*	0.835	0.933	0.782
	*K_F_* (mg·g^−1^)	13.06	4.63	4.89
	R^2^	0.899	0.925	0.937
Temkin	*K_T_* (L.mg^–1^)	0.210	0.090	0.065
	*b_T_* (J.mol^–1^)	18.509	15.92	23.73
	R^2^	0.757	0.743	0.813
D–R	*K_D_* (mol^2^J^–1^)	10^−5^	5 × 10^−5^	0.0001
	*q_m_* (mg·g^−1^)	249.011	235.003	209.013
	*E* (J.mol^−1^)	223.60	44.72	70.71
	R^2^	0.717	0.747	0.821

**Table 4:** Thermodynamic parameters for H_2_PO_4_^−^, HPO_4_^2−^, and NO_3_^−^ ion adsorption.

Ion	Δ*G*° (kJ/mol)				Δ*H*° (kJ/mol)	Δ*S*° (J/K/mol)
	25 °C	30 °C	35 °C	40 °C		
H_2_PO_4_^−^	−17.089	−18.436	−20.140	−25.038	134.603	506.40
HPO_4_^2−^	−14.85	−16.167	−17.178	−18.231	51.692	223.45
NO_3_^−^	−12.359	−14.138	−16.938	−21.291	161.32	581.45

**Table 5:** Comparison of maximum uptakes ($q_{m}$, mg/g) of various adsorbents to remove inorganic pollutants.

Adsorbent	Anions	*q_m_* (mg/g)	Reference
Raw shrimp shells	HPO_4_^2−^	92	[9]
	H_2_PO_4_^−^	133	[9]
Chitin	NO_3_^−^	200	[47]
	H_2_PO_4_^−^	336	[49]
	HPO_4_^2−^	110	[49]
*C. edulis* plant	H_2_PO_4_^−^	26.1	[65]
	NO_3_^−^	141.1	[65]
LDH/alginate composite	Phosphate	400	[27]
Alginate–Moroccan clay bio-nanocomposite	H_2_PO_4_^−^	625	This study
	HPO_4_^2−^	909.09	
	NO_3_^−^	588.23	

## Data Availability

Data are contained within the article.
